# Iterative Development and Usability Evaluation of an Open-Source Rehabilitation Follow-Up Platform: User-Centered Development Study

**DOI:** 10.2196/100614

**Published:** 2026-08-13

**Authors:** Noora Emilia Angelva, Franziska Gurschler, Michael Single, Valerie Frischknecht, Dominik Näf, Noel Kampus, Eva Cioffi, Thimo Marcin, Branislav Savic, Matthias Wilhelm, Anke Scheel-Sailer, Anna Lisa Martin-Niedecken, Urs Mosimann, Tobias Nef

**Affiliations:** 1 Gerontechnology and Rehabilitation Group, ARTORG Center for Biomedical Engineering Research, University of Bern, Freiburgstrasse 3, Bern, 3010, Switzerland, 41 782206577; 2 Graduate School for Health Sciences, University of Bern, Bern, Switzerland; 3 Centre for Rehabilitation and Sports Medicine, Inselspital Bern, University Hospital Bern, Bern, Switzerland; 4 Digital Health Design Living Lab, Institute for Design Research, Zurich University of the Arts, Zurich, Switzerland; 5 Department of Neurology, Inselspital Bern, University Hospital of Bern, Bern, Switzerland

**Keywords:** telerehabilitation, digital health, open-source software, usability evaluation, rehabilitation platform, patient self-management, user-centered design

## Abstract

**Background:**

Rehabilitation frequently extends beyond supervised clinical settings, creating a demand for scalable digital tools to support structured follow-up. We propose a novel open-source telerehabilitation platform.

**Objective:**

We describe the iterative, user-centered development of an open-source rehabilitation follow-up platform and report findings from an initial formative usability evaluation with patients, health care professionals, and additional end users.

**Methods:**

The platform was developed through repeated cycles of stakeholder engagement, prototyping, and evaluation within the rehabilitation department of Insel Group, University of Bern. A formative usability evaluation was conducted with 24 participants (8 health care professionals, 9 patients, and 7 additional participants; mean age 41.1, SD 12.9 y) using a task-based protocol. Perceived usability was assessed with the System Usability Scale (scored 0‐100) and the Post-Study System Usability Questionnaire (scored 1‐7; higher is better). Open-ended written responses were processed by one researcher using rapid content analysis.

**Results:**

A web-based telerehabilitation platform enables therapists to manage patient caseloads, assign and track personalized rehabilitation interventions, and monitor patient progress through Fitbit activity data and rehabilitation plan adherence. It supports multiclinic workflows with role-based access, REDCap patient importing (Vanderbilt University), automated background processing via Celery, and multilingual content. Its mean System Usability Scale score was 82.8 (SD 14.1), placing the platform in the “excellent” usability range. The mean Post-Study System Usability Questionnaire overall score was 6.02 (SD 0.77), with all 3 subscales scoring above 5.7. Most participants (20/24, 83%) rated the task set as easy or very easy; no participant rated any task as difficult. Among health care professionals, 7 out of 8 (88%) reported an intention to use the platform in practice. Qualitative themes highlighted the value of a rehabilitation plan overview, structured intervention scheduling, and progress tracking.

**Conclusions:**

The platform, released as open-source (Massachusetts Institute of Technology [MIT] License), demonstrated promising perceived usability across both patient and clinician user groups in a formative evaluation. These findings provide preliminary support for the platform’s perceived usability and justify further evaluation in real-world rehabilitation settings. Future work will evaluate deployment in real-world outpatient settings and assess clinical effectiveness.

## Introduction

The aging population is associated with an increase in chronic diseases, and population growth is placing pressure on health care systems [1]. Globally, the need for rehabilitation is increasing and remains substantially unmet, driven in part by population aging, chronic disease, and disability (Checklist 1)[2,3]. Rehabilitation often continues beyond the supervised clinical setting: to maintain functional gains and prevent complications, patients require structured exercise programs, tailored health information, and regular follow-up support after discharge. The resulting mismatch between limited clinical resources and the growing demand for long-term rehabilitation management underscores the need for scalable digital tools that bridge the gap between clinical discharge and sustained recovery at home.

Telerehabilitation has emerged as a promising approach for such tools, using telecommunications technologies to deliver rehabilitation services across geographic distances, supporting patient self-management and reducing travel barriers, particularly in rural or underserved areas [4-6]. Internet-based approaches have also demonstrated potential for cost savings by substituting for services that would otherwise be inaccessible [7]. Evidence from multiple systematic reviews indicates that telerehabilitation can achieve outcomes comparable to in-person rehabilitation across several rehabilitation contexts, including physical therapy broadly, Parkinson disease, and musculoskeletal disorders, and may be more effective than no rehabilitation in settings where face-to-face care is not feasible [5-8].

Well-designed telerehabilitation platforms can offer several advantages for clinical practice: they may enable clinicians to create and schedule individualized rehabilitation plans remotely, allow patients to access structured exercise instructions and educational materials at home, and provide longitudinal data on adherence and health parameters that can inform follow-up decisions [6,8,9]. By integrating wearable device data, patient-reported outcomes, and clinician-defined intervention schedules into a unified workflow, such platforms can support both clinical oversight and patient engagement, which are important for sustained rehabilitation outcomes [9,10].

However, platforms capable of delivering these benefits remain incompletely integrated into routine clinical use. A key barrier in digital health is vendor lock-in: proprietary solutions may create dependency on a single vendor’s technology, pricing, and product roadmap, limiting flexibility, portability, interoperability, and reproducibility in research [11]. Reviews of open-source clinical software and open-source electronic health record systems suggest that open-source development can enable local customization, support long-term sustainability, and foster collaborative development. However, adoption remains limited, and reporting is inconsistent [12,13]. More work is needed to translate the open-source paradigm into practical, clinically validated rehabilitation platforms.

To address these gaps, we developed an open-source rehabilitation follow-up platform guided by 3 principles: iterative, user-centered development; open-source transparency and reproducibility; and adaptability across diverse institutional and clinical contexts. The platform supports shared rehabilitation planning and patient self-management after discharge by integrating existing evidence-based rehabilitation content, structuring it into individualized plans, and providing clinicians and patients with a unified workflow for scheduling, monitoring, and feedback. It was developed at the Center for Rehabilitation & Sports Medicine (Inselspital and Berner Reha Zentrum), Bern University Hospital, using an approach that embedded clinical stakeholder input into each development cycle. Existing open-source platforms address related but distinct needs: OpenTera [14] provides a microservice architecture for rapid prototyping of telehealth and robotic applications, primarily in long-term care settings, but it does not focus on structured rehabilitation planning workflows. OpenTeleRehab [15], developed by Humanity & Inclusion and recognized as a Digital Public Good, offers treatment plan templates, an exercise library, and therapist-patient communication features, but it is designed primarily for deployment in low- and middle-income countries with limited digital infrastructure, including offline and SMS text messaging functionality. The proposed platform differs in its focus on integration with existing clinical workflows in high-resource hospital settings, its containerized architecture designed for institutional self-hosting under local data governance frameworks, its integration of wearable device data into structured rehabilitation plans, and its formal usability evaluation using validated instruments. Taken together, these distinctions define the space the platform is designed to fill: a formally evaluated, workflow-oriented, open-source platform for structured rehabilitation follow-up in high-resource hospital settings.

The platform’s core features were prioritized to address recognized barriers to rehabilitation follow-up, rather than solely for technical reasons. Limited adherence and engagement after discharge motivated structured scheduling, daily reminders, and visible progress feedback; discontinuity between inpatient and outpatient care motivated shared rehabilitation plans, an intervention library, and a unified clinician-patient workflow; and clinician workload motivated reusable plan templates, role-based caseload management, and consolidated patient overviews. Table 1 summarizes how each platform component is intended to address these barriers.

This paper makes 2 contributions: first, we describe the design and implementation of the platform as an open-source, modular web application; and second, we report findings from an initial formative usability evaluation conducted with patients, health care professionals, and additional end users within the rehabilitation department of the University Hospital of Bern [16].

**Table 1.** Conceptual mapping of barriers to rehabilitation follow-up and the platform components intended to address them.

Barrier to follow-up	Platform component intended to address it
Adherence or engagement	Scheduling, daily browser reminders, progress tracking, and “tiny-wins” feedback
Continuity of care	Shared rehabilitation plans, intervention library, and unified clinician-patient workflow
Clinician workload	Reusable plan templates, role-based caseload management, consolidated patient overview, and data export
Patient self-management	Patient dashboard, structured exercise and education content, and wearable-derived activity data

## Methods

### Design Approach and Iterative Development

We adopted an iterative, user-centered development approach informed by the IDEAS (integrate, design, assess, and share) framework [17]. Rather than following a linear development process, the platform was refined through repeated cycles of stakeholder input, rapid prototyping, implementation, and formative evaluation. In each cycle, feedback from patients, clinicians, and the research team was translated into concrete design requirements, which were implemented in the prototype and subsequently reviewed to identify usability issues, missing features, and opportunities for improvement. This process allowed the platform to evolve progressively in response to user needs and clinical workflow considerations.

Throughout these cycles, an interprofessional team (cardiology and sports medicine, rehabilitation medicine, neurology and neuropsychology, biomedical engineering and gerontechnology, exercise and rehabilitation science, and design research) met regularly to steer development and was supported by 3 structured co-design workshops. In the first workshop (8 team members; August 2024), candidate features were generated through brain-writing and ranked on a relevance-by-feasibility matrix. The priorities carried into development included a structured intervention library with expected benefits, goal-based intervention suggestions, progress tracking with motivating (“tiny wins”) feedback, and expert-approved constraints. These priorities shaped the rehabilitation-plan structure and intervention-metadata workflows. A second workshop with 3 patient representatives (October 2024) examined postdischarge needs: participants highlighted the loss of structure, reduced clinical support, and emotional strain, and called for a simple, accessible design with diagnosis-specific guidance and motivating feedback. When shown the team’s prioritized ideas, they endorsed them while cautioning that automated suggestions should complement rather than replace human feedback. A third workshop with patient representatives and clinicians (March 2025) compared 2 interaction concepts: a guided flowchart and a structured plan. Participants judged both useful at different recovery stages and stressed the importance of ease of use, web and mobile access, and data protection. These inputs underpin the barrier-to-feature mapping in Table 1 and the prototype evaluated here.

Stakeholder involvement was conducted in hospital clinics within the rehabilitation department of the University Hospital of Bern, engaging patients, clinicians, and researchers to identify needs and priorities for rehabilitation follow-up and digital support. Outputs from these activities informed core platform workflows (eg, rehabilitation plan structure, intervention metadata, and feedback capture) and prioritized features for successive development cycles.

Development proceeded through repeated cycles of (1) requirements elicitation and prioritization, (2) low- and high-fidelity prototyping, (3) implementation of prioritized features, and (4) formative evaluation and refinement. Users participated at each stage, as summarized in Table 2. The formal usability evaluation reported in this paper corresponds to one of these stages and was followed by an expert user experience (UX) review whose recommendations partly informed the current release.

**Table 2.** Iterative development phases, user involvement, and outputs^a^.

Phase	User involvement	Output
Requirements/needs elicitation	Patients, clinicians, and researchers	Core workflows: plan structure, intervention metadata, and feedback capture
Prototyping (low/high fidelity)	Stakeholder feedback on prototypes	Prioritized feature set and role-based interface designs
Formative usability evaluation (this study)	8 health care professionals, 9 patients, and 7 additional participants	SUS^b^/PSSUQ^c^ scores, task completion, and qualitative themes
Expert UX^d^ review	UX experts	Refinement priorities (landing page, navigation, dashboards, and motivation)
Postevaluation refinement	Implemented from review	For example, browser-based daily reminders (service worker) added to the current release

a The formative usability evaluation reported here represents one stage within this process.

b SUS: System Usability Scale.

c PSSUQ: Post-Study System Usability Questionnaire.

d UX: user experience.

### Platform Overview

The proposed platform is a modular web application designed for 2 primary user roles: patients and health care professionals. It provides role-based interfaces: a clinician-facing web portal to manage patients and rehabilitation plans and a patient-facing interface to view assigned interventions, respond to questionnaires, and review relevant health parameters (eg, activity or symptom tracking). Both interfaces are delivered as Progressive Web Apps, allowing them to be installed on mobile and desktop devices without requiring distribution through an app store. For patients, it includes a dashboard for quick status review and a calendar-based view of scheduled rehabilitation activities.

**Figure 1.**
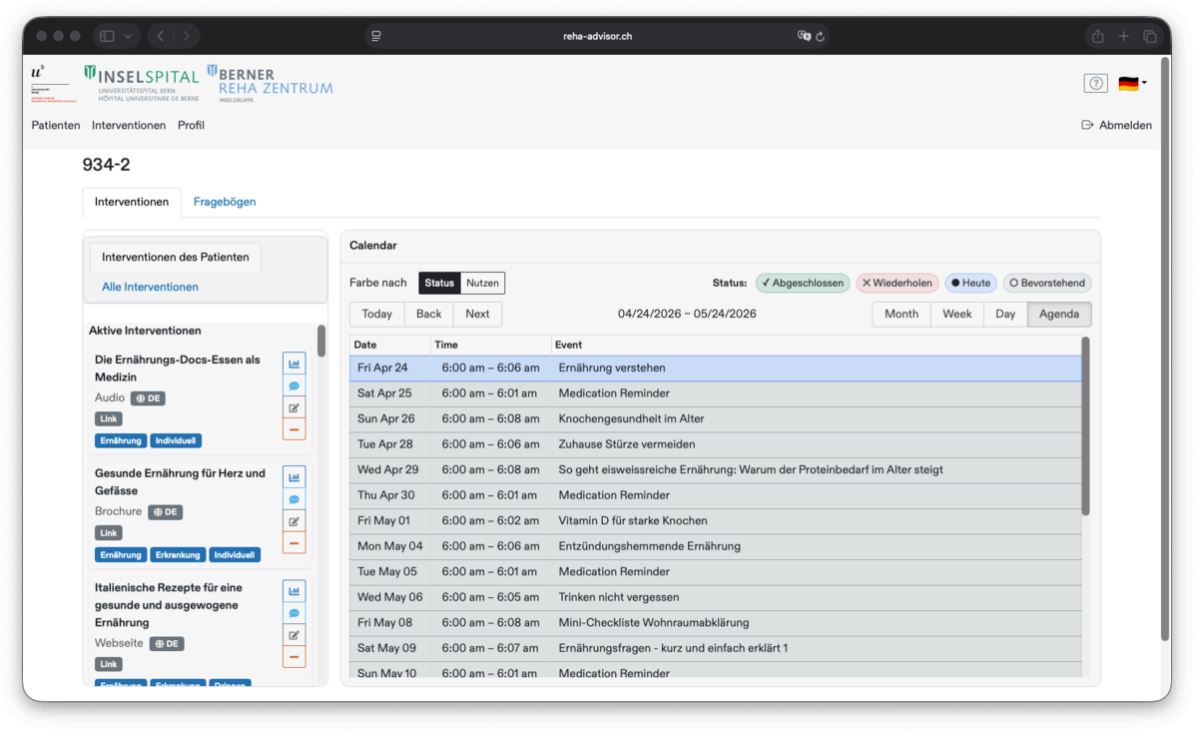
Clinical interface for intervention scheduling and monitoring. Calendar views (month/week/day/agenda) with status-coded interventions and access to the assigned intervention library.

**Figure 2.**
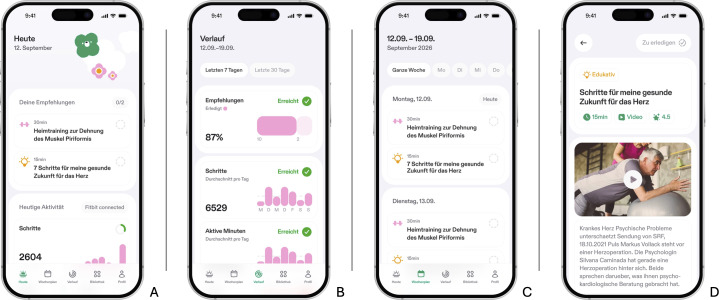
Patient interface for daily rehabilitation tasks and monitoring, shown across 4 screens of the patient-facing Progressive Web App. (A) Daily home screen ("Heute") showing the current date, the interventions recommended for that day, and today's synced activity data (eg, step count from a connected Fitbit device). (B) Intervention detail view for an individual educational ("Edukativ") intervention, including an embedded instructional video, which the patient can review and mark as completed. (C) Weekly plan view ("Wochenplan") listing the interventions scheduled across the days of the selected week. (D) Progress view ("Verlauf") summarizing longitudinal adherence and activity for a selected period (eg, the last 7 d), including the proportion of recommendations achieved, average daily step count, and active minutes, with status indicators showing which goals were met. Screen content is displayed in German, reflecting the platform's multilingual support.

Core functional modules include (1) user and role management; (2) patient administration; (3) an intervention library that supports searching, filtering, and creation of clinic-specific interventions; (4) rehabilitation plan templates and scheduling with calendar-based views; (5) patient feedback capture via questionnaires and postintervention feedback (including optional video feedback with time-limited storage); (6) data export for clinical documentation and research (eg, as PDFs or CSV files); and (7) daily notification reminders to support adherence, configurable through a dedicated settings interface. Figures 1 and 2 illustrate the end-to-end workflow from clinician planning to patient completion and feedback capture.

Rather than building all content from scratch, the platform is designed to integrate and curate existing evidence-based educational and rehabilitation resources (eg, publicly available exercise instructions, videos, or rehabilitation applications) and to enable local tailoring to patient needs. This strategy aims to reduce duplication and support the extension to additional rehabilitation contexts.

Rather than building all content from scratch, the platform is designed to integrate and curate existing evidence-based educational and rehabilitation resources (eg, publicly available exercise instructions, videos, or rehabilitation applications) and to enable local tailoring to patient needs. This strategy aims to reduce duplication and support extension to additional rehabilitation contexts.

### Technical Implementation and Architecture

The platform is based on a modular server-client architecture designed to support scalability, maintainability, reproducible deployment, and local adaptation. The front end is built with React 18 (Meta Platforms, Inc) and TypeScript (Microsoft Corp), using Vite for optimized build performance, and is delivered as a Progressive Web App. This enables installation on patients’ mobile and desktop devices directly from the browser and ensures the application functions reliably across varying network conditions. A service worker handles browser-based daily reminders that prompt patients to complete their assigned rehabilitation activities, using browser notification mechanisms compatible with the Web Notifications and Push API ecosystems [18]. Notification preferences are managed through a dedicated settings page and are compatible with browsers supporting the Web Notifications API. The back end is implemented with Django 5 (Django Software Foundation) and Django REST Framework (Encode OSS Ltd). Regarding data security, the back end employs token-based authentication, role-based access control, and encrypted data transport, with all data stored within the deploying institution’s own infrastructure to support data sovereignty.

For data management, the platform uses MongoDB (version 8.0.3; MongoDB, Inc) for flexible, document-based storage of interventions and patient records, alongside Redis (version 8.4.0; Redis Ltd) for in-memory caching and as a message broker for Celery background tasks. All services are served behind an NGINX (version 1.28.2; F5, Inc) reverse proxy, which handles static file serving, Secure Sockets Layer (SSL) termination, and request routing. The full stack is orchestrated with Docker (version 27.3.1; Docker, Inc), enabling reproducible deployments across different institutional IT infrastructures. The separation between the presentation layer, back-end services, background processing, and data storage allows individual components to be maintained, extended, or replaced independently. To support code quality and deployment confidence, the repository includes comprehensive test suites for both the front end (Jest, version 29.7.0; OpenJS Foundation, and React Testing Library, version 16.3.2) and the back end (Pytest, version 9.0.3), with continuous integration via GitHub Actions and automated coverage reporting.

For data management, the platform uses MongoDB (version 8.0.3) for flexible, document-based storage of interventions and patient records, alongside Redis (version 8.4.0) for in-memory caching and as a message broker for Celery background tasks. All services are served behind an NGINX (version 1.28.2) reverse proxy, which handles static file serving, SSL termination, and request routing. The full stack is orchestrated with Docker (version 27.3.1), enabling reproducible deployments across different institutional IT infrastructures. The separation between the presentation layer, back-end services, background processing, and data storage allows individual components to be maintained, extended, or replaced independently. To support code quality and deployment confidence, the repository includes comprehensive test suites for both the front end (Jest, version 29.7.0 and React Testing Library, version 16.3.2) and the back end (Pytest, version 9.0.3), with continuous integration via GitHub Actions and automated coverage reporting.

### Wearable Integration and Data Collection

To support longitudinal monitoring, the platform integrates with the Fitbit web service, which will be updated to the Google Health API due to the deprecation of the Fitbit web service in September 2026, via its publicly available REST API [19], which exposes activity, sleep, and physiological data from Fitbit wearable devices, using an OAuth 2.0 authorization flow [20]. Fitbit devices were selected because they operate independently of the user’s smartphone brand, lowering the barrier to access for patients. Once a patient authorizes access, the system performs daily automatic background synchronization.

The platform specifically retrieves and stores the following health metrics in the database:

- Physical activity: daily step counts, distance traveled, and active minutes categorized by heart rate zones
- Physiological data: resting and exercise heart rates
- Sleep metrics: total sleep duration and sleep quality indicators

These passive data streams are complemented by active patient reporting, including the manual entry of vital signs (eg, blood pressure and weight) and responses to structured questionnaires.

### Open-Source Strategy and Reproducibility

The software is released as open-source to support transparency, reuse, and reproducible research. The complete source code, documentation, and issue tracker are publicly available on GitHub and are released under the permissive Massachusetts Institute of Technology (MIT) License [16].

The proposed software’s open-source strategy is based on several principles: permissive licensing, public access to the codebase, modular software design, reproducible deployment, transparent issue tracking, and documentation for installation and further development. The modular architecture separates the front end, back end, background processing, and data storage components, allowing individual modules to be maintained, extended, or replaced independently. This design supports the platform’s adaptation to different rehabilitation contexts without requiring substantial changes to the overall system architecture.

Versioning of software artifacts is implemented using a Git-based repository structure. Source code, deployment files, documentation, and configuration artifacts, such as intervention templates or questionnaire definitions, when stored as repository files, can be tracked through commits, branches, release tags, and documented changes. This enables identifying the software state used in a given evaluation, reproducing deployments from a specific repository version, and tracing modifications over time. User-entered data are not versioned as source artifacts, but they can be exported in structured formats to support reproducibility, secondary analysis, and auditability. Stable software versions are documented using release tags, enabling specific platform versions to be referenced in publications, deployments, and evaluation studies.

To facilitate reuse across institutions, the repository includes developer documentation, deployment guidance, and contribution guidelines. These elements support external review, local adaptation, and collaborative development. Similar open-source strategies have been proposed to avoid reinventing existing components and to focus development efforts on extending capabilities and integrating reusable modules, as demonstrated by open-source telehealth frameworks such as OpenTera [14] and open-source telerehabilitation platforms such as OpenTeleRehab [15].

### Usability Evaluation: Participants and Recruitment

Participants were recruited through the rehabilitation department of the University Hospital of Bern (Inselspital) and the Berner Reha Zentrum in Heiligenschwendi, both of which are part of the Insel Group. The recruited sample included health care professionals from the rehabilitation department, patients with rehabilitation experience, and additional healthy participants. Inclusion criteria were (1) aged 18 years or older and (2) the ability to provide written informed consent. Participants were not required to have prior experience with the platform.

The sample size was chosen pragmatically, in line with the evaluation’s formative purpose. For early-stage usability testing, small samples are commonly used to identify recurring usability problems and inform iterative refinement. The Nielsen usability testing heuristic suggests that testing approximately 5 users can reveal many major usability issues within a user group, particularly when combined with iterative design cycles [21]. In this study, the inclusion of 24 participants, comprising 8 health care professionals, 9 patients, and 7 additional participants, was therefore considered appropriate for assessing perceived usability and identifying design improvements across the main intended user perspectives. For quantitative usability scoring with the System Usability Scale (SUS), this sample size also provides a pragmatic basis for estimating overall perceived usability, although group-specific estimates remain descriptive because of the limited number of participants per subgroup [22]. The study was not powered to test clinical effectiveness or to conduct inferential comparisons between participant groups.

The evaluation assessed 2 role-specific interfaces. Patients evaluated the patient-facing interface and health care professionals evaluated the clinician-facing interface, reflecting their respective intended uses. The additional participant group evaluated both interfaces, providing cross-interface usability coverage and a larger per-interface denominator (patient interface: n=16; clinician interface: n=15). This approach is appropriate for early-stage formative testing, where the priority is to surface recurring interface-level usability problems efficiently rather than to obtain population-representative estimates. Accordingly, we report SUS and Post-Study System Usability Questionnaire (PSSUQ) outcomes for health care professionals and patients as the primary user populations and present the additional participant group as supplementary, exploratory data.

### Formative Usability Protocol

The usability evaluation was conducted on a prototype of the rehabilitation platform that included core workflows for rehabilitation planning, scheduling, patient task completion, and feedback. Features implemented after this evaluation are described separately as subsequent design refinements and were not included in the usability scores reported here. The formative evaluation used a moderated, task-based usability protocol to assess the software’s core platform workflows for both user roles. Sessions were conducted in person at the rehabilitation department of the University Hospital of Bern (Inselspital) between August 2025 and December 2025. Before starting the tasks, participants received a short verbal introduction explaining the purpose of the evaluation, the voluntary nature of participation, and that the study aimed to evaluate the platform rather than the participants’ performance. Participants were allowed to ask clarification questions before the task session started. During task completion, however, they were asked to work independently and without assistance, unless technical access problems required assistance.

Participants completed the tasks using study-provided hardware or their own devices. The platform was tested on computers and/or smartphones, reflecting the intended use of the clinician-facing and patient-facing interfaces. Clinician tasks were primarily performed on laptop devices, whereas patient-facing tasks were performed on smartphones, depending on participant availability and device preference. No strict maximum time limit was imposed, as the aim was to observe task completion and perceived usability under realistic formative conditions rather than to assess speed under time pressure. Task completion and notable usability issues were observed by the first author and/or the second author using a standardized task checklist.

Participants were asked to complete the following standardized task sets:

- Patient interface tasks: logging in, viewing the interventions scheduled for the current day, marking an intervention as completed, and submitting feedback after it is completed.
- Clinician interface tasks: creating a user account, logging in, assigning a recommendation to a patient with a given diagnosis, creating a new patient profile and a rehabilitation plan with 3 interventions, creating an intervention and assigning it to patient groups, and editing user profile information.

No formal think-aloud protocol was used. Instead, participants were encouraged to report difficulties or uncertainties if they occurred, and the observer documented task completion, usability issues, and spontaneous comments. After completing the tasks and exploring the platform, participants completed standardized usability questionnaires and a written semistructured posttask questionnaire.

The questionnaire combined closed-ended ratings with open-ended questions to capture perceived task difficulty, prior experience with digital technologies, perceived usefulness, barriers to use, and suggestions for improvement. Health care professionals received additional role-specific questions on expected usefulness in rehabilitation workflows, intention to use, perceived effects on time and workload, real-time monitoring, trust in platform-generated recommendations, and willingness to recommend the platform. The complete posttask questionnaire, including the standardized usability instruments and the role-specific open-ended items, is provided as Multimedia Appendix 1.

The written questionnaire format was used to standardize data collection across participants and to minimize interviewer influence. Responses were entered into a structured electronic case report form. Because the open-ended responses were collected in written form, no audio recording or verbatim transcription was required. The questionnaire guide is provided in Multimedia Appendix 1.

### Outcome Measures

Perceived usability was assessed using the SUS (10 items, 1‐5 response scale, scored 0‐100) [23] and the PSSUQ (16 items, 1‐7 response scale) [24]. The SUS was scored according to the standard scoring procedure, with positively worded items scored as the item score minus 1, and negatively worded items scored as 5 minus the item score; the sum was then multiplied by 2.5 to obtain a score from 0 to 100.

For the PSSUQ, the original positively worded item content was retained. However, the response direction was aligned with the SUS and the other agreement-based items administered during the same usability assessment session. Specifically, the electronic questionnaire used a consistent agreement scale in which lower values indicated disagreement and higher values indicated agreement. This was done to reduce the risk of response errors caused by switching between opposite response directions across questionnaires within the same assessment. As a result, PSSUQ scores in this study are reported in a study-specific higher-is-better direction, rather than in the conventional PSSUQ lower-is-better direction. For comparability with conventional PSSUQ reporting, nonmissing item scores can be transformed using *x*_standard_=8 *− x*_study_. Responses coded as 99 were treated as missing. The full mapping is provided in Multimedia Appendix 2.

Additional questions assessed perceived task difficulty (1=“very easy” to 5=“very difficult”), and among health care professionals, perceived usefulness for follow-up care, intention to use, perceived effect on time and workload, perceived usefulness for real-time monitoring, trust requirements for platform recommendations, and willingness to recommend the platform. Open-ended questions captured positive aspects, barriers, missing functions, and suggested areas for improvement.

### Statistical Analysis

We conducted descriptive analyses for SUS and PSSUQ scores overall and by participant group (including health care professionals, patients, and additional participants). Continuous scores are reported as mean and SD. Missing items were treated as missing. SUS scores were calculated only when all 10 items were completed. PSSUQ scale scores were computed as the mean of available items after excluding responses coded as 99 (not applicable). PSSUQ scores are reported in the study-specific direction, where higher values indicate better perceived usability. For transparency and comparability with conventional PSSUQ scoring, the conversion rule to the standard lower-is-better direction is reported in Multimedia Appendix 2. Task completion was summarized as the number and proportion of participants who completed each standardized task, computed separately for the patient-facing and clinician-facing interfaces. Perceived task difficulty was summarized as counts by response category, overall, and by group. The study was not powered for inferential comparisons between groups, and no hypothesis tests were performed.

### Qualitative Analysis

Open-ended responses from the written posttask questionnaire were analyzed using rapid content analysis, an approach suited to formative and implementation-oriented evaluations that require timely identification of actionable themes [25]. Responses were exported from REDCap (Vanderbilt University) and organized in a structured matrix by question topic and participant group using Microsoft Excel. One researcher coded the responses, grouping them into recurring categories (perceived strengths, usability issues, missing functions, and improvement suggestions) and consolidating these into overarching themes; representative quotations were then selected to illustrate each theme across participant groups. The analysis focused on identifying actionable findings for the next development iteration rather than on generating a formal qualitative theory, and no formal interrater reliability statistic was computed. The limitation arising from single-researcher coding is addressed in the *Limitations* section.

### Ethical Considerations

All procedures performed in the study involving human participants were conducted in accordance with the ethical standards of the institutional and/or national research committee and with the Declaration of Helsinki and its later amendments [26]. Written informed consent was obtained from all participants before study participation. This study was approved by the Bern Cantonal Ethics Committee (KEK Bern: 2025‐01071).

## Results

### Participants

In total, 24 participants completed the usability evaluation, including 8 health care professionals, 9 patients, and 7 additional participants. Participant characteristics are summarized in Table 3.

**Table 3.** Participant characteristics (N=24).

Characteristic	Overall (N=24)	Health care professionals (n=8)	Patients (n=9)	Other (n=7)
Female, n (%)	10 (41.7)	5 (62.5)	1 (11.1)	4 (57.1)
Male, n (%)	14 (58.3)	3 (37.5)	8 (88.9)	3 (42.9)
Age (y), mean (SD; range)	41.1 (12.9; 25-63)	33.9 (6.7; 27-44)	54.3 (8.5; 36-63)	32.4 (8.1; 25-48)

### Usability Outcomes

Overall perceived usability was high. The mean SUS score was 82.8 (SD 14.1). The mean PSSUQ overall score, reported in the study-specific higher-is-better direction, was 6.02 (SD 0.77), corresponding to 1.98 in the conventional lower-is-better PSSUQ direction. Subscale results are shown in Table 4.

**Table 4.** Usability outcomes (N=24)^a^.

Outcome (scale)	Overall (N=24), mean (SD)	Health care professionals (n=8), mean (SD)	Patients (n=9), mean (SD)	Regular (n=7), mean (SD)
SUS^b^ (0‐100)	82.8 (14.1)	79.4 (13.7)	83.1 (16.3)	86.4 (12.5)
PSSUQ^c^ overall (1-7; higher=better, study direction)	6.02 (0.77)	5.74 (0.94)	6.23 (0.76)	6.06 (0.57)
System usefulness (items 1‐6)	6.12 (0.80)	5.88 (0.92)	6.19 (0.87)	6.31 (0.56)
Information quality (items 7‐12)	5.92 (0.88)	5.50 (1.17)	6.29 (0.61)	5.94 (0.65)
Interface quality (items 13‐15)	5.95 (0.74)	5.81 (0.70)	6.15 (0.85)	5.86 (0.69)

a PSSUQ scores are reported in the study-specific higher-is-better direction. To convert to the conventional PSSUQ lower-is-better direction, use *x*_standard_=8*−x*_study_ for each nonmissing item.

b SUS: System Usability Scale.

c PSSUQ: Post-Study System Usability Questionnaire.

Most participants rated the task set as easy or very easy (20/24, 83%); the remaining participants reported neutral difficulty (4/24, 17%). No participant rated the tasks as difficult or very difficult. Perceived difficulty was consistent across groups: 6 out of 8 (75%) health care professionals, 7 out of 9 (78%) patients, and 7 out of 7 (100%) additional participants rated the platform as easy or very easy to use, with the remainder rating it as neutral.

Among 8 health care professionals, 7 (88%) reported that they would use the platform in practice. Six health care professionals reported that they would recommend it to colleagues, one was unsure, and one response was missing. This corresponds to 6 out of 8 (75%) of all health care professionals or 6 out of 7 (86%) of those with valid responses. The perceived impact on workload was mixed: 3 out of 8 reported that the platform would reduce time and workload, 1 out of 8 reported that it would not, 3 out of 8 were unsure, and 1 response was missing. Most health care professionals (6/8, 75%) reported that real-time monitoring features would be helpful. Among patients, 7 out of 9 (78%) found the rehabilitation platform easy or very easy to use, while 2 out of 9 (22%) reported neutral difficulty.

### Task Completion

Task completion was high across both interfaces. All participants who attempted a task completed it, and no task failures were recorded against the standardized checklist (Table 5). The 2 patient-interface tasks were completed by all 16 participants (9 patients and 7 additional participants). The 6 clinician-interface tasks were completed by all participants (8 health care professionals and 7 additional participants); for the account-creation task, 1 record was missing, yielding 14 of 15 documented completions. Per-task details are provided in Multimedia Appendix 3. Because the evaluation used realistic, untimed conditions without a think-aloud protocol, time on task, error counts, and help requests were not systematically quantified; observed difficulties were documented descriptively and incorporated into the qualitative themes.

**Table 5.** Task completion by interface^a^.

Task	Interface	Completed, n/N (%)
Log in and view interventions scheduled for today	Patient	16/16 (100)
Mark an intervention as completed and submit feedback	Patient	16/16 (100)
Create a user account	Clinician	14/14 documented (1 missing)^b^
Log in	Clinician	15/15 (100)
Assign a recommendation to a patient with a given diagnosis	Clinician	15/15 (100)
Create a patient profile and rehabilitation plan with 3 interventions	Clinician	15/15 (100)
Create an intervention and assign it to patient groups	Clinician	15/15 (100)
Edit user profile information	Clinician	15/15 (100)

a Patient-interface tasks were performed by patients and additional participants (n=16); clinician-interface tasks were performed by health care professionals and additional participants (n=15).

b One clinician-interface participant had no recorded result for this task (treated as missing). No task was recorded as failed.

### Qualitative Feedback Analysis

#### Overview

Overall, the qualitative feedback was consistent with the high-quantitative usability ratings while highlighting specific points of friction. The reported frustrations are therefore interpreted as actionable usability issues identified during formative testing rather than as contradicting the positive usability scores.

Four recurring themes were identified, summarized below; representative quotations and their cross-group distribution are provided in Multimedia Appendix 4.

#### Value of Overview and Progress Tracking

Participants appreciated having an at-a-glance view of rehabilitation plans and daily activities, as well as the ability to review health and activity data over time, such as steps, sleep, and vital parameters. Several participants requested clearer progress indicators, such as “tiny wins,” improved visualization of changes over time, and more actionable interpretation of collected parameters. This was the most frequently raised theme and was expressed across all 3 participant groups. One patient noted that “the overall picture is very useful” [Gesamtbild ist sehr nützlich], while others requested clearer progress feedback, for example, “a weekly overview; e.g. 80% achieved” (German: “Wochenübersicht; bspw. 80% erreicht”) and, from an additional participant, “progress for the patients—tiny wins!”

#### Intervention Library and Structured Planning

Health care professionals valued the ability to create standard plans and templates, search and explore interventions, and schedule activities. Patients valued receiving structured exercise instructions and educational content. Several participants expressed interest in communication features, such as in-app messaging, and in tailoring interventions more explicitly to individual rehabilitation goals. One patient valued that “exercises are provided, and you do not have to assemble a program yourself” (German: “Gut, dass Übungen da sind *…”*), and an additional participant wished to “chat with my therapist through it.”

#### Interface Simplification and Consistency

Suggested improvements included simplifying screens with many buttons, making views more compact, improving the calendar and navigation, and standardizing terminology and visual design. Some participants reported frustration with error messages and session handling (eg, unexpected logouts). This was the second-most-frequently raised theme. One health care professional observed that there were “currently still very many buttons; the system needs to be faster to operate” (German: “*…*sehr viele Buttons *…*”), and friction with error handling and session management was noted directly, for example, “sudden log out” and “bad error messages.”

#### Trust and Clinical Validation

Participants emphasized that trust depends on clinician-validated content, transparent sourcing of educational materials, and clarity about who selected or approved interventions. Health care professionals noted the importance of integrating the platform into established clinical workflows and ensuring that recommendations and content are verified before patient release. This theme was raised predominantly by health care professionals; one emphasized the need for content “always confirmed by a therapist before being released” (German: *“…*von einem Therapeuten bestätigt *…”*), and an additional participant valued content that was “[personalised] from my therapist (approved).”

### Design Refinements Identified Through UX Review

In parallel with the formative usability testing, an expert UX review workshop was conducted to identify priorities for subsequent design refinement. The usability scores reported in this study, therefore, reflect the prototype version available during the evaluation, not all the features included in the current open-source release.

During usability testing, the evaluated prototype included the core clinician and patient workflows, including user log in, patient profile creation, intervention library access, rehabilitation plan creation, scheduled intervention assignment, patient-facing daily intervention views, completion marking, and postintervention feedback submission. The expert UX review focused on how these workflows could be improved in subsequent iterations.

Key recommendations included adding a concise landing page that clearly communicates the platform’s purpose, streamlining the sitemap and navigation for both user roles, providing dashboards for at-a-glance status information, unifying patient data representations, and improving patient-profile views by combining plan adherence, questionnaire completion, and recent activity. The review also highlighted the potential value of notification support and motivational elements, such as visualization of the rehabilitation journey or gamification, to sustain patient engagement.

Some of these recommendations were implemented after the formative evaluation. In particular, browser-based daily reminders using a service worker were added after usability testing and are therefore part of the current platform version, but they were not reflected in the reported SUS and PSSUQ scores. Other recommendations, such as more advanced motivational elements and extended dashboard visualizations, remain priorities for future development.

## Discussion

### Principal Findings

This was a formative, single-session usability evaluation. It assessed perceived usability and task completion under short, moderated, task-based conditions and did not assess adherence, workflow burden, long-term engagement, clinical effectiveness, or real-world adoption; the following interpretations are bounded accordingly.

The rehabilitation platform achieved a mean SUS score of 82.8 (SD 14.1), placing it in the “excellent” usability range according to established SUS adjective-rating benchmarks [27]. The mean PSSUQ overall score was 6.02 (SD 0.77) on a 7-point scale, with all 3 subscales (system usefulness, information quality, and interface quality) scoring above 5.7. Scores were consistent across participant groups, with patients rating both instruments marginally higher than health care professionals did, suggesting that the patient-facing interface was particularly well received. Crucially, 20 out of 24 (83%) participants rated the task set as easy or very easy, and no participant rated any task as difficult.

The qualitative findings provide complementary clinical insight. The features participants valued most—at-a-glance rehabilitation plan overview, longitudinal progress tracking, and structured intervention scheduling—map onto broader telerehabilitation implementation priorities, including adherence, engagement, accessibility, satisfaction, and integration into routine workflows [8,10,28]. In our formative data, participants specifically emphasized the importance of visible progress and interpretable feedback. The repeated interest in “tiny wins” and actionable feedback may also be interpreted through self-determination theory, particularly the role of perceived competence in supporting sustained motivation and health behavior change [29]. Together, these themes suggest that the platform’s design priorities were appropriate to the clinical context.

Among 8 health care professionals, 7 (88%) reported an intention to use the platform in clinical practice, and 6 out of 8 (75%) would recommend it to colleagues; these are encouraging early signals that require confirmation in deployment studies. The mixed workload impact ratings (3/8 expecting workload reduction and 3/8 uncertain) are expected for a formative prototype and point to specific areas requiring workflow optimization before deployment, particularly plan template creation and assignment workflows. The strong interest in real-time monitoring features (6/8, 75%), combined with the security and content validation concerns raised in qualitative feedback, underscores the importance of clear data governance communication and clinician-controlled content approval workflows in the next development iteration.

### Comparison With Prior Work

The usability scores reported here add to an evidence base in which many telerehabilitation studies primarily emphasize clinical, physiological, adherence, or algorithmic outcomes. In contrast, detailed reporting of role-specific UX and formative usability findings is less consistently described [6,8,9,30]. The observed SUS score above 80 and the PSSUQ overall score above 6.0 suggest strong perceived usability at the formative stage. The consistency across patient and health care professional groups further suggests that the role-differentiated interface design did not introduce major usability asymmetries that would complicate deployment. These findings provide early empirical support for the feasibility of a workflow-oriented, open-source approach in this domain. For comparison, we focus on 2 commonly reported strands of digital rehabilitation research: telemonitoring systems and AI-focused rehabilitation tools. These areas were selected because they represent 2 major directions in current rehabilitation technology development, both directly relevant to the platform’s intended scope. Telemonitoring systems typically focus on remote observation, physiological data capture, exercise supervision, or adherence monitoring [9,30]. AI-focused rehabilitation tools, in contrast, often emphasize prediction, activity recognition, movement assessment, or decision-support models [31-33]. Both strands are highly relevant to future rehabilitation services, but they often address specific functional components rather than the broader workflow infrastructure needed to plan, assign, monitor, and adapt rehabilitation interventions across patient and clinician roles. To make the platform’s intended niche and its contribution relative to the state of the art more explicit, Table 6 contrasts the platform with the open-source systems introduced earlier (OpenTera and OpenTeleRehab) and with these 2 strands across capability, setting, deployment, and evaluation dimensions.

Compared with commonly reported telemonitoring systems and AI-focused rehabilitation tools, the proposed platform emphasizes workflow infrastructure. The platform does not aim to replace remote monitoring systems or specialized AI models; rather, it provides an extensible environment in which such components can be embedded. Its current contribution lies in supporting intervention libraries, structured rehabilitation planning and scheduling, patient feedback capture, progress documentation, and data export. In this sense, the platform complements existing telemonitoring and AI-oriented approaches by addressing the organizational and interaction layers required to integrate digital rehabilitation tools into clinical workflows.

**Table 6.** Structured comparison of the proposed platform with related approaches^a^.

Approach	Primary focus	Target setting/context	Rehabilitation workflow support	Data/wearable integration	Openness and deployment model	Evaluation status/evidence base	Relationship to proposed platform
Proposed platform	Open-source infrastructure for structured rehabilitation follow-up: plan management, patient self-management, and clinician-patient workflow support	High-resource hospital and outpatient rehabilitation settings; designed for institutional self-hosting	Intervention library, clinician-assigned plans, scheduling, patient task completion, postintervention feedback, progress documentation, and data export	Integrates Fitbit-derived activity and physiological data; supports manual patient-reported data and questionnaires	Open-source (MIT^b^ License); containerized modular architecture for local institutional deployment under local data-governance frameworks	Formative, single-session usability evaluation (n=24; SUS^c^, PSSUQ^d^, task completion, qualitative feedback); adherence, long-term use, and clinical effectiveness not yet assessed	— (reference point for the comparison)
OpenTera [14]	Microservice architecture for rapid prototyping of telehealth and robotic applications	Care-facility and long-term-care contexts (originally a COVID-19 response)	General telehealth application framework oriented toward flexible prototyping rather than structured rehabilitation-plan management	Extensible architecture; wearable/Fitbit integration is not a described focus	Open-source telehealth framework	Published description of architecture and use cases [14]; not assessed in this study	A broader telehealth/robotics prototyping framework; complementary, as rehabilitation-planning workflows of the type provided here could be built on such an architecture
OpenTeleRehab [15]	Open-source telerehabilitation platform with treatment-plan templates, an exercise library, and therapist-patient communication	Primarily low- and middle-income countries and low-resource settings with limited digital infrastructure	Treatment-plan templates and communication features, with emphasis on low-infrastructure delivery (offline/SMS text messaging)	Optimized for low-infrastructure use (offline/SMS text messaging); wearable integration is not a described focus	Open-source; recognized as a Digital Public Good with documented real-world field deployment	Established field deployment as a Digital Public Good [15]; not assessed in this study	Complementary, with different design priorities: OpenTeleRehab optimizes for low-resource and offline contexts, whereas this platform targets self-hosted integration into high-resource hospital workflows with wearable-supported follow-up
Telemonitoring systems (research category)	Remote observation, physiological-data capture, exercise supervision, and/or adherence monitoring	Often disease- or program-specific remote rehabilitation contexts	Typically monitor activity, symptoms, or adherence; less commonly provide an integrated plan-assign-adapt-document workflow across patient and clinician roles	Frequently sensor-, device-, or physiological-data oriented	Varies across systems; often system-specific or proprietary	Individual systems report clinical, physiological, adherence, or monitoring outcomes (eg, [9,30])	Strong at generating follow-up data; this platform focuses on the organizational workflow layer needed to use such data within rehabilitation planning and could incorporate telemonitoring outputs
AI-focused rehabilitation tools (research category)	Prediction, activity recognition, movement assessment, or decision-support models	Often research prototypes or specialized digital rehabilitation modules	Usually address specific analytical or decision-support tasks rather than full patient-clinician workflow management	Often use sensor, video, wearable, or task-performance data depending on the model	Varies across tools; may be proprietary, experimental, or model-specific	Individual studies report algorithmic or model-performance outcomes (eg, [31-33])	This platform is not itself primarily an AI system; it provides extensible workflow infrastructure into which such models could be embedded as future modules

a OpenTera and OpenTeleRehab are specifically named systems, whereas “telemonitoring systems” and “AI-focused rehabilitation tools” are broad research categories included to situate the platform relative to 2 major directions in rehabilitation technology. For these 2 categories, the cells provide illustrative, nonexhaustive characterizations drawn from the cited examples rather than a systematic survey. Cells describe each approach’s primary design orientation; the absence of a feature denotes a different design focus, not a judged deficiency.

b MIT: Massachusetts Institute of Technology.

c SUS: System Usability Scale.

d PSSUQ: Post-Study System Usability Questionnaire.

### The Value of Open-Source Code in Rehabilitation Technology

A central contribution of this work is to release the software’s source code under the permissive MIT License. This directly addresses the widespread issue of vendor lock-in in digital health, which systematic reviews have identified as a barrier to sustainable adoption and cross-institutional collaboration [12,13]. By making the entire codebase publicly available, we enable independent evaluation, adaptation, and extension by other research groups and clinical institutions without licensing constraints.

The containerized, modular architecture enables institutions to host the platform locally, supporting institutional data governance and compliance with applicable national frameworks, such as Swiss human research legislation [34], while contributing improvements to the shared code base. In addition to packaging the software components themselves, container-based deployment supports the explicit management of runtime state through persistent volumes. Volumes can store data generated and used by running services, such as database contents, uploaded media, configuration files, logs, or study-specific artifacts. When appropriately documented, anonymized, and governed, such volumes or volume exports can be archived and shared with a specific software version to reproduce complex study states, for example, a configured study instance, test dataset, or demonstration environment.

This distinction between container images and persistent volumes is relevant for reproducible rehabilitation research. Container images define the executable software environment, whereas volumes preserve selected runtime state across container restarts, updates, or redeployments. In research contexts, it is important to distinguish between sharing the platform implementation, sharing a configured deployment, and sharing study artifacts. Sensitive runtime data, such as patient records or study recordings, should not be included in public releases unless fully anonymized and ethically approved for sharing. Instead, public repositories can provide empty volume definitions, synthetic example data, documented import/export procedures, and where appropriate, restricted-access volume snapshots for reproducibility audits.

This positions the software as an extensible infrastructure rather than a finished product, a distinction that aligns with the iterative development philosophy described in the *Methods* section. Institutions can adopt the platform and adapt it to local clinical workflows, regulatory requirements, and patient populations without dependency on a single vendor’s roadmap or pricing decisions.

An open-source release also supports reproducible research: other groups can inspect the implementation, replicate the evaluation, and build on the platform for their own clinical studies. This is particularly important in a field where proprietary systems often prevent independent verification of technical claims and limit cross-site comparability.

### Implications of an Open-Source Rehabilitation Platform

Open-source development in digital health enhances transparency, reproducibility, and long-term sustainability by allowing stakeholders to inspect, modify, and extend software independently of vendor constraints. Reviews of open-source clinical software and electronic health record systems emphasize both opportunities and challenges, including variability in project maturity, documentation standards, and governance models [12,13]. Nevertheless, open-source ecosystems can reduce vendor lock-in, foster collaborative development, and support local adaptation, particularly in heterogeneous rehabilitation environments.

Rehabilitation workflows vary substantially across institutions, specialties, and patient populations. Proprietary systems may limit configurability and integration with external tools. A modular open-source platform can mitigate these constraints by enabling shared intervention libraries, standardized data structures, structured feedback capture, and extensible interfaces for integration with external services. In this context, the platform’s architecture supports integration with wearable device ecosystems, including Fitbit-based data exchange, enabling the incorporation of activity, sleep, and physiological metrics into structured rehabilitation follow-up. By separating core infrastructure (eg, user management, scheduling, and documentation export) from optional analytics and wearable integration modules, the proposed platform supports incremental extension without requiring monolithic deployment.

Beyond technical transparency, open-source architectures are intended to facilitate reproducible digital health research by enabling independent verification, replication studies, and cross-institutional reuse—areas that are frequently constrained by proprietary rehabilitation platforms. Cross-institutional reuse was not evaluated in this study.

### Limitations and Future Work

This study reflects an early-stage formative usability evaluation and has several limitations.

First, the sample size was modest, and participants were recruited through the rehabilitation department of the University Hospital of Bern and affiliated rehabilitation settings, which may limit generalizability to broader rehabilitation populations and other institutional contexts. Second, participants reported relatively high familiarity with digital technologies, which may have contributed to favorable usability ratings. Third, the evaluation assessed perceived usability during short task-based sessions and did not measure long-term engagement, adherence, clinical outcomes, or integration into routine rehabilitation pathways. Fourth, the qualitative analysis of open-ended responses was conducted by one researcher. Although the analysis was structured around predefined categories and focused on identifying actionable usability issues for iterative development, the absence of independent dual coding or a formal interrater reliability assessment may have introduced interpretive bias. Future evaluations should include independent coding by multiple researchers or consensus-based qualitative analysis to strengthen the credibility of qualitative findings.

Future studies should include longitudinal field trials in real-world outpatient settings, assessment of sustained engagement and adherence, and evaluation of clinical effectiveness and workflow integration. Scalability under real-world load conditions, including concurrent multiuser access and high-volume data synchronization, has not yet been tested and should be evaluated in deployment studies.

Further development will focus on refinement, clinical integration, technical extension of the platform, and incorporation of feedback from the UX workshop. We will prioritize UX improvements, including simplifying navigation, optimizing the calendar, improving onboarding workflows, providing clearer progress summaries, and enhancing error handling, to further improve usability. Browser-based daily notification reminders have already been implemented in the current version.

Additional motivational features are planned to support adherence during longitudinal follow-up. These may include visualization of the rehabilitation journey, progress indicators, goal-setting functions, completion streaks, milestone badges, and feedback on small achievements or “tiny wins.” Such features should be designed carefully to support motivation without creating pressure, guilt, or inappropriate competition among patients. In rehabilitation contexts, gamification may therefore benefit from focusing on self-monitoring, encouragement, and personalized progress feedback rather than on competitive ranking. Future studies should examine whether these motivational components improve adherence, patient engagement, and perceived self-efficacy over time.

Subsequent work will evaluate the platform’s deployment in real-world outpatient rehabilitation workflows, including the assessment of sustained engagement, adherence patterns, and its impact on patient-reported and clinical outcomes. Implementation studies will examine barriers and facilitators to institutional adoption, including training requirements, governance processes, and integration with existing information systems.

From a technical perspective, future extensions will include deeper integration of wearable device ecosystems via standardized interfaces, such as Fitbit, enabling automated import of activity, sleep, and physiological data into structured rehabilitation plans. The platform architecture is designed to support the integration of AI-based motor assessment and decision-support modules while preserving modularity and reproducibility. Interoperability testing with electronic health record systems using standardized data exchange protocols, such as Fast Healthcare Interoperability Resources, will further support clinical integration and scalability [35].

### Conclusions

This study describes the iterative development and formative usability evaluation of the platform, an open-source rehabilitation follow-up tool designed to support structured plan management, patient self-management, and clinician-patient workflow integration. In contrast to monitoring-centric telehealth systems and algorithm-focused AI rehabilitation tools, the platform emphasizes reproducible infrastructure, modular extensibility, and structured rehabilitation planning within real-world follow-up contexts.

The high usability scores observed in this formative evaluation suggest that an open-source, workflow-oriented rehabilitation platform can achieve strong perceived usability among both patients and health care professionals. By integrating structured intervention libraries, standardized feedback capture, and extensible interfaces for wearable device ecosystems, the platform is intended to provide a foundation for scalable, interoperable rehabilitation follow-up; this potential remains to be evaluated in deployment studies.

A central contribution of this work is to release the platform source code under the MIT License, directly addressing the widespread issue of vendor lock-in in digital health. By providing a containerized, modular architecture (Django or React or MongoDB), the architecture can help institutions host the platform locally, supporting data sovereignty and long-term research reproducibility. Future work will focus on longitudinal deployment within outpatient rehabilitation workflows and the evaluation of sustained engagement and clinical effectiveness.

## Supplementary material

10.2196/100614 Multimedia Appendix 1 Usability task protocol and posttask questionnaire.

10.2196/100614 Multimedia Appendix 2 Post-Study System Usability Questionnaire response direction and scoring.

10.2196/100614 Multimedia Appendix 3 Task completion detail.

10.2196/100614 Multimedia Appendix 4 Qualitative theme–quotation matrix.

10.2196/100614 Checklist 1 Complete REDCap data collection instrument for the Reha-Advisor usability and acceptance study (L2025_Reha-Advisor SUE). The bilingual (German/English) booklet comprises participant eligibility and demographic forms, a role-specific task-performance checklist for health care professionals and patients, two validated usability questionnaires (PSSUQ, System Usability Scale), a semistructured interview guide covering perceived usefulness, trust, and adoption intention, and end-of-study documentation.

## Abbreviations

IDEAS: integrate, design, assess, and share
KEK: Kantonale Ethikkommission (Cantonal Ethics Committee)
MIT: Massachusetts Institute of Technology
PSSUQ: Post-Study System Usability Questionnaire
SSL: Secure Sockets Layer
SUS: System Usability Scale
UX: user experience
