# Supplementary material for: Iterative Development and Usability Evaluation of an Open-Source Rehabilitation Follow-Up Platform: User-Centered Development Study
Source: JMIR Rehabil Assist Technol. 2026 Aug 13;13:e100614. doi: 10.2196/100614 (PMC13473804; doi:10.2196/100614)
Supplement: Supplementary file 1 [file rehab-v13-e100614-s005.pdf]

# CTU Template Eligibility

Record ID __________________________________

Participant ID __________________________________

Informed Consent Form signed by participant ◯ Yes ◯ No

Date of Informed Consent Form signature by participant __________________________________

Version of Informed Consent Form signed __________________________________
(e.g. 1.0)

Date of the Informed Consent Form version __________________________________

Consent Form for further use of data and biological material signed by participant ◯ Yes ◯ No

Date of consent for further use by participant __________________________________

Gender
◯ male
◯ female

Year of Birth __________________________________

Age at inclusion (calculated) __________________________________

# Task Performance

| | |
|---|---|
| Subject type | ◯ Health Care Professional<br>◯ Patient<br>◯ Regular Participant |
| Log in and view your interventions for today. (Patient) | ◯ True<br>◯ False |
| Mark an intervention as completed and answer feedback. (Patient) | ◯ True<br>◯ False |
| Create your user (Not for Patient) | ◯ True<br>◯ False |
| Log in (Not for Patient) | ◯ True<br>◯ False |
| Assign a new recommendation to a patient with diagnosis X. (Not for Patient) | ◯ True<br>◯ False |
| Create a patient X and create their rehabilitation plan with 3 interventions. (Not for Patient) | ◯ True<br>◯ False |
| Create an intervention and assign to patient groups. (Not for Patient) | ◯ True<br>◯ False |
| Change your information. (Not for Patient) | ◯ True<br>◯ False |

# PSSUQ

## Systemnützlichkeit (SYSUSE) / System Usefulness (SYSUSE)

| | |
|---|---|
| Insgesamt bin ich damit zufrieden, wie einfach dieses System zu bedienen ist.<br><br>* System bedeutet die Plattform<br>(Overall, I am satisfied with how easy it is to use this system.) | ◯ Stimme überhaupt nicht zu<br>◯ Stimme nicht zu<br>◯ Stimme eher nicht zu<br>◯ Weder zustimmen noch ablehnen<br>◯ Stimme eher zu<br>◯ Stimme zu<br>◯ Stimme voll und ganz zu<br>◯ N.A. |
| Dieses System war einfach zu bedienen.<br><br>*System bedeutet die Plattform<br>(It was simple to use this system.) | ◯ Stimme überhaupt nicht zu<br>◯ Stimme nicht zu<br>◯ Stimme eher nicht zu<br>◯ Weder zustimmen noch ablehnen<br>◯ Stimme eher zu<br>◯ Stimme zu<br>◯ Stimme voll und ganz zu<br>◯ N.A. |
| Mit diesem System konnte ich die Aufgaben und Szenarien schnell erledigen<br><br>*System bedeutet die Plattform<br>** Aufgaben und Szenarien beziehen sich auf das Einloggen und Ausprobieren der Funktionen der Plattform<br>(I was able to complete the tasks and scenarios quickly using this system.) | ◯ Stimme überhaupt nicht zu<br>◯ Stimme nicht zu<br>◯ Stimme eher nicht zu<br>◯ Weder zustimmen noch ablehnen<br>◯ Stimme eher zu<br>◯ Stimme zu<br>◯ Stimme voll und ganz zu<br>◯ N.A. |
| Ich habe mich mit diesem System wohl gefühlt.<br><br>*System bedeutet die Plattform<br>(I felt comfortable using this system.) | ◯ Stimme überhaupt nicht zu<br>◯ Stimme nicht zu<br>◯ Stimme eher nicht zu<br>◯ Weder zustimmen noch ablehnen<br>◯ Stimme eher zu<br>◯ Stimme zu<br>◯ Stimme voll und ganz zu<br>◯ N.A. |
| Die Verwendung dieses Systems war leicht zu erlernen.<br><br>*System bedeutet die Plattform<br>(It was easy to learn to use this system.) | ◯ Stimme überhaupt nicht zu<br>◯ Stimme nicht zu<br>◯ Stimme eher nicht zu<br>◯ Weder zustimmen noch ablehnen<br>◯ Stimme eher zu<br>◯ Stimme zu<br>◯ Stimme voll und ganz zu<br>◯ N.A. |
| Ich glaube, dass ich mit diesem System schnell produktiv werden kann.<br><br>*System bedeutet die Plattform<br>(I believe I could become productive quickly using this system.) | ◯ Stimme überhaupt nicht zu<br>◯ Stimme nicht zu<br>◯ Stimme eher nicht zu<br>◯ Weder zustimmen noch ablehnen<br>◯ Stimme eher zu<br>◯ Stimme zu<br>◯ Stimme voll und ganz zu<br>◯ N.A. |

## Informationsqualität (INFOQUAL) / Information Quality (INFOQUAL)

| | |
|---|---|
| Das System gab Fehlermeldungen aus, die mir deutlich sagten, wie ich Probleme beheben kann.<br><br>*System bedeutet die Plattform<br>(The system gave error messages that clearly told me how to<br>fix problems.) | ◯ Stimme überhaupt nicht zu<br>◯ Stimme nicht zu<br>◯ Stimme eher nicht zu<br>◯ Weder zustimmen noch ablehnen<br>◯ Stimme eher zu<br>◯ Stimme zu<br>◯ Stimme voll und ganz zu<br>◯ N.A. |
| Wenn mir bei der Verwendung des Systems ein Fehler unterlief,<br>konnte ich ihn leicht und schnell beheben.<br><br>*System bedeutet die Plattform<br>(Whenever I made a mistake using the system, I could recover<br>easily and quickly.) | ◯ Stimme überhaupt nicht zu<br>◯ Stimme nicht zu<br>◯ Stimme eher nicht zu<br>◯ Weder zustimmen noch ablehnen<br>◯ Stimme eher zu<br>◯ Stimme zu<br>◯ Stimme voll und ganz zu<br>◯ N.A. |
| Die Informationen (z. B. Online-Hilfe, Bildschirmmeldungen und sonstige Dokumentation), die mit diesem System bereitgestellt werden, waren eindeutig.<br><br>*System bedeutet die Plattform<br>(The information (such as online help, on-screen messages, and other documentation) provided with this system was clear.) | ◯ Stimme überhaupt nicht zu<br>◯ Stimme nicht zu<br>◯ Stimme eher nicht zu<br>◯ Weder zustimmen noch ablehnen<br>◯ Stimme eher zu<br>◯ Stimme zu<br>◯ Stimme voll und ganz zu<br>◯ N.A. |
| Es war einfach, die benötigten Informationen zu finden.<br><br>*System bedeutet die Plattform<br>(It was easy to find the information I needed.) | ◯ Stimme überhaupt nicht zu<br>◯ Stimme nicht zu<br>◯ Stimme eher nicht zu<br>◯ Weder zustimmen noch ablehnen<br>◯ Stimme eher zu<br>◯ Stimme zu<br>◯ Stimme voll und ganz zu<br>◯ N.A. |
| Die Informationen haben mir bei der Bewältigung der Aufgaben und Szenarien sehr geholfen.<br><br>* Aufgaben und Szenarien beziehen sich auf das Einloggen und Ausprobieren der Funktionen der Plattform<br>(The information was effective in helping me complete the tasks and scenarios.) | ◯ Stimme überhaupt nicht zu<br>◯ Stimme nicht zu<br>◯ Stimme eher nicht zu<br>◯ Weder zustimmen noch ablehnen<br>◯ Stimme eher zu<br>◯ Stimme zu<br>◯ Stimme voll und ganz zu<br>◯ N.A. |
| Die Anordnung der Informationen auf den Bildschirmen des Systems war klar.<br><br>*System bedeutet die Plattform<br>(The organization of information on the system screens was clear.) | ◯ Stimme überhaupt nicht zu<br>◯ Stimme nicht zu<br>◯ Stimme eher nicht zu<br>◯ Weder zustimmen noch ablehnen<br>◯ Stimme eher zu<br>◯ Stimme zu<br>◯ Stimme voll und ganz zu<br>◯ N.A. |

## Schnittstellenqualität (INTERQUAL) / Interface Quality (INTERQUAL)

| | |
|---|---|
| Die Gestaltung der Benutzeroberfläche spricht mich an.<br><br>*Der Benutzeroberfläche bedeutet die Plattformschnittstelle<br>(The interface of this system was pleasant.) | ◯ Stimme überhaupt nicht zu<br>◯ Stimme nicht zu<br>◯ Stimme eher nicht zu<br>◯ Weder zustimmen noch ablehnen<br>◯ Stimme eher zu<br>◯ Stimme zu<br>◯ Stimme voll und ganz zu<br>◯ N.A. |
| Ich benutze das System gerne.<br><br>* System bedeutet die Plattform<br>(I liked using the interface of this system.) | ◯ Stimme überhaupt nicht zu<br>◯ Stimme nicht zu<br>◯ Stimme eher nicht zu<br>◯ Weder zustimmen noch ablehnen<br>◯ Stimme eher zu<br>◯ Stimme zu<br>◯ Stimme voll und ganz zu<br>◯ N.A. |
| Dieses System hat alle Funktionen und Möglichkeiten, die ich erwarte.<br><br>*System bedeutet die Plattform<br>(This system has all the functions and capabilities I expect it to have.) | ◯ Stimme überhaupt nicht zu<br>◯ Stimme nicht zu<br>◯ Stimme eher nicht zu<br>◯ Weder zustimmen noch ablehnen<br>◯ Stimme eher zu<br>◯ Stimme zu<br>◯ Stimme voll und ganz zu<br>◯ N.A. |
| Insgesamt bin ich mit diesem System zufrieden.<br><br>*System bedeutet die Plattform<br>(Overall, I am satisfied with this system.) | ◯ Stimme überhaupt nicht zu<br>◯ Stimme nicht zu<br>◯ Stimme eher nicht zu<br>◯ Weder zustimmen noch ablehnen<br>◯ Stimme eher zu<br>◯ Stimme zu<br>◯ Stimme voll und ganz zu<br>◯ N.A. |

# Interview

Date of the interview

______________________________

Version/Test Platform

______________________________

Auf einer Skala von 1 bis 5: Wie sicher fühlen Sie sich im Umgang mit digitalen Technologien im Allgemeinen?

(On a scale from 1 to 5, how confident do you feel in using digital technologies in general?)

______________________________

Könnten Sie uns etwas mehr über Ihre Erfahrungen mit digitalen Technologien erzählen? Welche Geräte oder Apps nutzen Sie am häufigsten?

(Could you tell us a bit more about your experience with digital technologies? What kinds of devices or apps do you use most often?)

______________________________

HCP only
Auf einer Skala von 1 bis 5: Wie sicher fühlen Sie sich bei der Verwendung digitaler Technologien im Rahmen der Patientenrehabilitation?

(On a scale from 1 to 5, how confident do you feel in using digital technology as part of patient rehabilitation?)

______________________________

HCP only
In welchen Situationen fühlen Sie sich am wohlsten bzw. am unwohlsten, wenn Sie Technologie in der Rehabilitation einsetzen?

(What are some situations where you feel most comfortable or least comfortable using technology in rehabilitation?)

______________________________

Wie einfach oder schwierig fanden Sie die Nutzung der Plattform insgesamt?

(How easy or difficult did you find the overall platform to use?)

- ◯ Sehr einfach
- ◯ Einfach
- ◯ Neutral
- ◯ Schwierig
- ◯ Sehr schwierig

Welche Aufgaben (falls vorhanden) fanden Sie verwirrend oder schwer zu erledigen?

* Aufgaben und Szenarien beziehen sich auf das Einloggen und Ausprobieren der Funktionen der Plattform
(Which tasks (if any) did you find confusing or hard to complete?)

______________________________

Gab es bestimmte Teile der Plattform, die sich besonders reibungslos oder efficient bedienen liessen?

(Were there any specific parts of the platform that felt particularly smooth or efficient to use?)

__________________________________

---

Hat Sie etwas bei der Nutzung der Plattform gestört?

(Did anything frustrate you while using the platform?)

__________________________________

---

Gibt es Ihrer Meinung nach etwas, das auf der Plattform fehlt, beispielsweise eine Funktion?

(Is there something misisng in the platform in your opinion, such as a function?)

__________________________

---

HCP Only
Glauben Sie, dass diese Plattform Ihnen bei Ihren täglichen Rehabilitationsaufgaben helfen könnte?

(Do you feel this platform could be helpful in your daily rehab tasks?)

- ◯ Überhaupt nicht
- ◯ Etwas
- ◯ Neutral
- ◯ Hilfreich
- ◯ Sehr hilfreich

---

HCP Only
Würden Sie diese Plattform regelmässig nutzen, wenn es vollständig verfügbar wäre?

(Would you use this plarform regularly if it were fully available?)

- ◯ Nein
- ◯ Vielleicht
- ◯ Ja

---

Welche Funktionen wären für Sie am nützlichsten?

(What features would be most useful to you?)

__________________________________

---

Was würden Sie ändern oder verbessern?

(What would you change or improve?)

__________________________________

---

HCP Only
Glauben Sie, dass der Plattform die Zeit reduzieren kann, die für die Zuweisung oder Aktualisierung von Rehabilitationsplänen benötigt wird?

(Would you see the platform being able to reduce the time needed to assign or update rehabilitation plans?)

- ◯ Ja
- ◯ Nein
- ◯ Ich bin mir nicht sicher

HCP Only
Würde Ihnen eine Echtzeit-Patientenüberwachung helfen, effektiver einzugreifen?

(Would real-time patient monitoring help you intervene more effectively?)

◯ Ja
◯ Nein

---

HCP Only
Was würde Sie dazu bewegen, der Vorschlägen der Plattforms für Interventionen oder Anpassungen zu vertrauen?

(What would make you trust the paltform's suggestions for interventions or adjustments?)

__________________________________

---

HCP Only
Würden Sie diese Plattform Kollegen oder anderen Patienten weiterempfehlen?

(Would you recommend this platform to colleagues or other patients?)

◯ Ja
◯ Nein
◯ Ich bin mir nicht sicher

---

Motivation zur Nutzung der Plattform. Sollte es eine Art Belohnung für die Nutzung und die Durchführung der zugewiesenen Interventionen geben? Wenn ja, wie würde diese aussehen?

(Motivation to use the platform. Should there be some kind of rewards for using it and completing the assigned interventions? if so what would the rewards be?)

__________________________________

---

Haben Sie noch weitere Rückmeldungen, die Sie uns mitteilen möchten?

(Any other feedback you would like to share?)

__________________________________

# System Usability Survey

| | |
|---|---|
| Ich denke, dass ich das System gerne häuftig benutzen würde.<br><br>* System bedeutet die Plattform<br>(I think that I would like to use this system frequently.) | ◯ 1 Stimme überhaupt nicht zu (Strongly Disagree)<br>◯ 2<br>◯ 3<br>◯ 4<br>◯ 5 Stimme voll und ganz zu (Strongly Agree) |
| Ich fand das System unnötig komplex.<br><br>* System bedeutet die Plattform<br>(I found the system unnecessarily complex.) | ◯ 1 Stimme überhaupt nicht zu (Strongly Disagree)<br>◯ 2<br>◯ 3<br>◯ 4<br>◯ 5 Stimme voll und ganz zu (Strongly Agree) |
| Ich fand das System einfach zu benutzen.<br><br>* System bedeutet die Plattform<br>(I thought the system was easy to use.) | ◯ 1 Stimme überhaupt nicht zu (Strongly Disagree)<br>◯ 2<br>◯ 3<br>◯ 4<br>◯ 5 Stimme voll und ganz zu (Strongly Agree) |
| Ich glaube, ich würde die Hilfe einer technisch versierten Person benötigen, um das System benutzen zu<br>können.<br><br>* System bedeutet die Plattform<br>(I think that I would need the support of a technical person to be able to use this system.) | ◯ 1 Stimme überhaupt nicht zu (Strongly Disagree)<br>◯ 2<br>◯ 3<br>◯ 4<br>◯ 5 Stimme voll und ganz zu (Strongly Agree) |
| Ich fand, die verschiedenen Funktionen in diesem System waren gut integriert.<br><br>* System bedeutet die Plattform<br>(I found the various functions in this system were well integrated.) | ◯ 1 Stimme überhaupt nicht zu (Strongly Disagree)<br>◯ 2<br>◯ 3<br>◯ 4<br>◯ 5 Stimme voll und ganz zu (Strongly Agree) |
| Ich denke, das System enthielt zu viele Inkonsistenzen.<br><br>* System bedeutet die Plattform<br>** Inkonsistenzen bedeuten, dass die Funktionen oder das Design der Plattform nicht einheitlich waren<br>(I thought there was too much inconsistency in this system.) | ◯ 1 Stimme überhaupt nicht zu (Strongly Disagree)<br>◯ 2<br>◯ 3<br>◯ 4<br>◯ 5 Stimme voll und ganz zu (Strongly Agree) |
| Ich kann mir vorstellen, dass die meisten Menschen den Umgang mit diesem System sehr schnell lernen.<br><br>* System bedeutet die Plattform<br>(I would imagine that most people would learn to use this system very quickly.) | ◯ 1 Stimme überhaupt nicht zu (Strongly Disagree)<br>◯ 2<br>◯ 3<br>◯ 4<br>◯ 5 Stimme voll und ganz zu (Strongly Agree) |
| Ich fand das System sehr umständlich zu nutzen.<br><br>* System bedeutet die Plattform<br>(I found the system very cumbersome to use.) | ◯ 1 Stimme überhaupt nicht zu (Strongly Disagree)<br>◯ 2<br>◯ 3<br>◯ 4<br>◯ 5 Stimme voll und ganz zu (Strongly Agree) |

| | |
|---|---|
| Ich fühlte mich bei der Benutzung des Systems sehr sicher.<br><br>* System bedeutet die Plattform<br>(I felt very confident using the system.) | ◯ 1 Stimme überhaupt nicht zu (Strongly Disagree)<br>◯ 2<br>◯ 3<br>◯ 4<br>◯ 5 Stimme voll und ganz zu (Strongly Agree) |
| Ich musste eine Menge lernen, bevor ich anfangen konnte das System zu verwenden.<br><br>* System bedeutet die Plattform<br>(I needed to learn a lot of things before I could get going with this system.) | ◯ 1 Stimme überhaupt nicht zu (Strongly Disagree)<br>◯ 2<br>◯ 3<br>◯ 4<br>◯ 5 Stimme voll und ganz zu (Strongly Agree) |

# CTU Template End of Study

Patient has completed the study according to protocol? ◯ Yes ◯ No

Completion Date ________________________

Reason for non-completion
◯ Patient withdrew consent
◯ Patient was lost to follow-up
◯ Patient died
◯ Other reason
