# Supplementary material for: Iterative Development and Usability Evaluation of an Open-Source Rehabilitation Follow-Up Platform: User-Centered Development Study
Source: JMIR Rehabil Assist Technol. 2026 Aug 13;13:e100614. doi: 10.2196/100614 (PMC13473804; doi:10.2196/100614)
Supplement: Supplementary file 2 [file rehab-v13-e100614-s001.docx]

# Appendix 1: Usability Task Protocol and Post-task Questionnaire

## Pre-task Instructions

Before starting the usability tasks, participants received the following verbal instructions:

Thank you for taking part in this usability evaluation. The aim of this session is to evaluate the proposed platform, not your performance. We are interested in whether the platform is understandable, useful, and easy to use. Please complete the following tasks as independently as possible. You may ask clarification questions before starting. During the tasks, the study team will not provide assistance unless there is a techni- cal problem that prevents you from continuing. After the tasks, you will be asked to complete usability questionnaires and provide written feedback.

## Task Checklist

Table 3: Standardised usability task checklist.

**User role	Tasks**

Patient interface	Log in and view interventions scheduled for today; mark an interven- tion as completed; answer post-intervention feedback.

Clinician interface	Create a user account; log in; assign a recommendation to a patient with a specified diagnosis; create a patient profile and rehabilitation plan with three interventions; create an intervention and assign it to patient groups; change user profile information.

## Written Post-task Questionnaire

The following questions were used in the written semi-structured post-task questionnaire:

1. On a scale from 1 to 5, how confident do you feel in using digital technologies in general?
2. Could you tell us more about your experience with digital technologies? Which devices or apps do you use most often?
3. Health care professionals only: On a scale from 1 to 5, how confident do you feel using digital technologies as part of patient rehabilitation?
4. Health care professionals only: In which situations do you feel most comfortable or least comfortable using technology in rehabilitation?
5. How easy or difficult did you find the overall platform to use?
6. Which tasks, if any, did you find confusing or difficult to complete?
7. Were there any parts of the platform that felt particularly smooth or efficient to use?
8. Did anything frustrate you while using the platform?
9. Is anything missing from the platform, such as a function or feature?
10. Health care professionals only: Do you feel this platform could be helpful in your daily rehabilitation tasks?
11. Health care professionals only: Would you use this platform regularly if it were fully avail- able?
12. Which features would be most useful to you?
13. What would you change or improve?
14. Health care professionals only: Could the platform reduce the time needed to assign or update rehabilitation plans?
15. Health care professionals only: Would real-time patient monitoring help you intervene more effectively?
16. Health care professionals only: What would make you trust the platform’s suggestions for interventions or adaptations?
17. Health care professionals only: Would you recommend this platform to colleagues or pa- tients?
18. Should there be any form of motivation or reward for using the platform and completing assigned interventions? If yes, what should it look like?
19. Do you have any other feedback?
