# Supplementary material for: Iterative Development and Usability Evaluation of an Open-Source Rehabilitation Follow-Up Platform: User-Centered Development Study
Source: JMIR Rehabil Assist Technol. 2026 Aug 13;13:e100614. doi: 10.2196/100614 (PMC13473804; doi:10.2196/100614)
Supplement: Supplementary file 3 [file rehab-v13-e100614-s002.docx]

# Appendix 2: PSSUQ Response Direction and Scoring

The PSSUQ was administered using the original positively worded item content but with reversed response coding compared with the conventional PSSUQ format. This adaptation was made because the PSSUQ was administered together with the SUS and other agreement-based study items. A consistent response direction was used throughout the questionnaire battery to reduce participant confusion and potential response errors. In this study, 1 indicated strongest disagreement and 7 indicated strongest agreement; therefore, higher values indicate better perceived usability.

Table 4: PSSUQ response coding used in this study and conversion to the conventional PSSUQ direction.

| **Study response code** | **Study label** | **Conventional PSSUQ-equivalent code** |
| --- | --- | --- |
| 1 | Strongly disagree | 7 |
| 2 | Disagree | 6 |
| 3 | Somewhat disagree | 5 |
| 4 | Neither agree nor disagree | 4 |
| 5 | Somewhat agree | 3 |
| 6 | Agree | 2 |
| 7 | Strongly agree | 1 |
| 99 | Not applicable | Missing |

Conventional PSSUQ-equivalent scores can be obtained by applying:

*x*standard = 8 *− x*study

to each non-missing item.
