# Supplementary material for: Iterative Development and Usability Evaluation of an Open-Source Rehabilitation Follow-Up Platform: User-Centered Development Study
Source: JMIR Rehabil Assist Technol. 2026 Aug 13;13:e100614. doi: 10.2196/100614 (PMC13473804; doi:10.2196/100614)
Supplement: Supplementary file 4 [file rehab-v13-e100614-s003.docx]

# Appendix 3: Task Completion Detail

Task completion was recorded on a standardised checklist during each moderated session. Completion was recorded affirmatively; a non-completed or abandoned task would have been recorded as not completed. No task was recorded as not completed during the evaluation. Patient-interface tasks were performed by patients (n=9) and additional participants (n=7); clinician-interface tasks were performed by health care professionals (n=8) and additional participants (n=7). For the account-creation task, one clinician-interface participant had no recorded result, which was treated as missing. Time on task, error counts, and help requests were not systematically quantified, consistent with the realistic, untimed, non-think-aloud formative protocol; observed difficulties were documented descriptively and incorporated into the qualitative themes. Per-task completion is reported in Table 5.
