# Supplementary material for: Iterative Development and Usability Evaluation of an Open-Source Rehabilitation Follow-Up Platform: User-Centered Development Study
Source: JMIR Rehabil Assist Technol. 2026 Aug 13;13:e100614. doi: 10.2196/100614 (PMC13473804; doi:10.2196/100614)
Supplement: Supplementary file 5 [file rehab-v13-e100614-s004.docx]

# Appendix 4: Qualitative Theme–Quotation Matrix

Quotations are participant-provided responses to the written post-task questionnaire (translated to English; German originals in brackets). Group tags: HCP = health care professional; P = patient; A = additional participant.

| **Theme (cross-group distribution)** | **Group** | **Representative quotation** |
| --- | --- | --- |
| Value of overview and progress tracking (all groups; most frequently raised) | P | The overall picture is very useful. [*Gesamtbild ist sehr nützlich*] |
|  | A | Progress for the patients — tiny wins! |
|  | P | Weekly overview; e.g. 80% achieved. [*Wochenübersicht; bspw. 80% erreicht*] |
|  | HCP | Patients should be able to view their own progress over recent days and weeks. |
| Intervention library and structured planning (HCP, patients, additional) | P | Good that exercises are provided and you do not have to assemble a programme yourself. |
|  | HCP | Exercise collections from many therapists are visible. |
|  | A | Chat with my therapist through it. |
| Interface simplification and consistency (all groups; second most frequent) | HCP | Currently still very many buttons; the system needs to be faster to operate. |
|  | HCP | Sudden log out; not always clear what the task does. |
|  | A | Bad error messages and patient edit portal. |
| Trust and clinical validation (predominantly HCP) | HCP | Content should always be confirmed by a therapist before being released. |
|  | A | [Personalised] from my therapist (approved). |
|  | HCP | Coherent algorithms tailored to diagnosis and patient preferences. |
